# Transplantation of Adult Mouse iPS Cell-Derived Photoreceptor Precursors Restores Retinal Structure and Function in Degenerative Mice

**DOI:** 10.1371/journal.pone.0018992

**Published:** 2011-04-29

**Authors:** Budd A. Tucker, In-Hyun Park, Sara D. Qi, Henry J. Klassen, Caihui Jiang, Jing Yao, Stephen Redenti, George Q. Daley, Michael J. Young

**Affiliations:** 1 Department of Ophthalmology, Institute for Vision Research, Carver College of Medicine, University of Iowa, Iowa City, Iowa, United States of America; 2 Department of Ophthalmology, Schepens Eye Research Institute, Harvard Medical School, Boston, Massachusetts, United States of America; 3 Division of Pediatric Hematology/Oncology, Children's Hospital Boston and Dana Farber Cancer Institute, Boston, Massachusetts, United States of America; 4 Department of Biological Chemistry and Molecular Pharmacology, Harvard Medical School, Boston, Massachusetts, United States of America; 5 Manton Center for Orphan Disease Research, Harvard Stem Cell Institute, Howard Hughes Medical Institute, Children's Hospital Boston, Boston, Massachusetts, United States of America; 6 Department of Ophthalmology, School of Medicine, Gavin Herbert Eye Institute, University of California Irvine, Orange, California, United States of America; 7 Department of Ophthalmology, Eye&ENT Hospital, Shanghai Medical School, Fudan University, Shanghai, China; Seattle Children's Research Institute, United States of America

## Abstract

This study was designed to determine whether adult mouse induced pluripotent stem cells (iPSCs), could be used to produce retinal precursors and subsequently photoreceptor cells for retinal transplantation to restore retinal function in degenerative hosts. iPSCs were generated using adult dsRed mouse dermal fibroblasts via retroviral induction of the transcription factors Oct4, Sox2, KLF4 and c-Myc. As with normal mouse ES cells, adult dsRed iPSCs expressed the pluripotency genes SSEA1, Oct4, Sox2, KLF4, c-Myc and Nanog. Following transplantation into the eye of immune-compromised retinal degenerative mice these cells proceeded to form teratomas containing tissue comprising all three germ layers. At 33 days post-differentiation a large proportion of the cells expressed the retinal progenitor cell marker Pax6 and went on to express the photoreceptor markers, CRX, recoverin, and rhodopsin. When tested using calcium imaging these cells were shown to exhibit characteristics of normal retinal physiology, responding to delivery of neurotransmitters. Following subretinal transplantation into degenerative hosts differentiated iPSCs took up residence in the retinal outer nuclear layer and gave rise to increased electro retinal function as determined by ERG and functional anatomy. As such, adult fibroblast-derived iPSCs provide a viable source for the production of retinal precursors to be used for transplantation and treatment of retinal degenerative disease.

## Introduction

Retinal degenerative diseases such as retinitis pigmentosa (RP) and age-related macular degeneration (AMD) are currently the leading cause of incurable blindness in the western world [1], [2], [3]. These diseases are characterized by death of the light sensing photoreceptor cells of the outer neural retina. As the intrinsic regenerative capacity of the mammalian retina is extremely limited, the only viable treatment option for people suffering from photoreceptor cell loss is cellular replacement.

Over the past decade, stem/progenitor cell transplantation as a means of inducing tissue reconstruction and functional regeneration has garnered extensive interest in the field of regenerative medicine. Within the retina in particular, many exciting advances have been made. One significant achievement came in 2004 when a subset of transplanted retinal progenitor cells was shown to develop into a variety of mature retinal neurons, including retinal ganglion and photoreceptor cells [4]. Since then, numerous studies reporting varying degrees of success have utilized an assortment of different cell types ranging from the fate-restricted photoreceptor precursor [5] to the pluripotent embryonic stem (ES) cell [6], [7], [8], [9]. ES cells in particular are of interest due to their ability to undergo unlimited expansion and subsequent tissue specific differentiation. These inherent properties may allow one to generate a sufficiently large number of cells in order to perform clinical transplantation from single isolations rather than requiring multiple new donations, as is potentially the problem when using more terminally differentiated cell types. However, like photoreceptor precursor and retinal progenitor cells, issues pertaining to host donor compatibility and ethics of cellular isolation exist (i.e. human versions of the above mentioned cells are isolated during embryonic development). Thus, generation/selection of a cell type more suited for clinical application would be desirable.

A cell type that avoids such problems is the recently generated, induced pluripotent stem cell (iPSC). Initially produced by Takahashi and Yamanaka (2006), iPSCs were generated via genetic reprogramming of dermal fibroblasts to pluripotency using retroviral transduction with the four transcription factors Oct4, Sox2, KLF4 and c-Myc [10]. Although the originally published protocols remain the most effective and efficient means of inducing pluripotency in adult dermal fibroblasts, a variety of new protocols with variations in cell type, delivery methods and reprogramming factors have been developed [11], [12], [13], [14], [15], [16], [17], [18], [19]. Such variations are aimed at reducing the need for virally induced genetic insertion of the potentially tumorogenic factors c-Myc and KLF4. In doing so, it has become evident that reprogramming is exponentially more efficient, requiring less genetic manipulation, when cells isolated from developmentally immature tissues are used. For instance, unlike fibroblasts isolated from adult skin, embryonic fibroblasts have been successfully reprogrammed using the transcription factors OCT4 and Sox2 alone [20]. However, as retinal degenerative disorders are in large part diseases of adulthood, to be clinically relevant derivation of patient specific iPSCs from accessible adult tissue will be required.

As demonstrated using the above mentioned cell types, production of iPSC derived retinal cells expressing a variety of retinal proteins, including those specific to photoreceptors, have been achieved [21], [22]. Furthermore it has recently shown that retinal neurons produced from human iPSCs can engraft the mouse retina following delivery to the subretinal space [23]. However, to date the safety and capacity for functional integration following ocular transplantation have yet to be investigated. In this study, iPSCs were generated from adult dsRed-mouse dermal fibroblasts, differentiated toward retinal photoreceptor precursors, and transplanted into retinal degenerative hosts. Rod and cone photoreceptor cell repopulation, synapse formation and cellular integration associated with restoration of electrophysiological and anatomical correlates of retinal function were observed. These findings establish a proof-of-principle for applications of autologous iPSCs for the safe and effective treatment of retinal degenerative diseases.

## Results and Discussion

**Figure 1:**
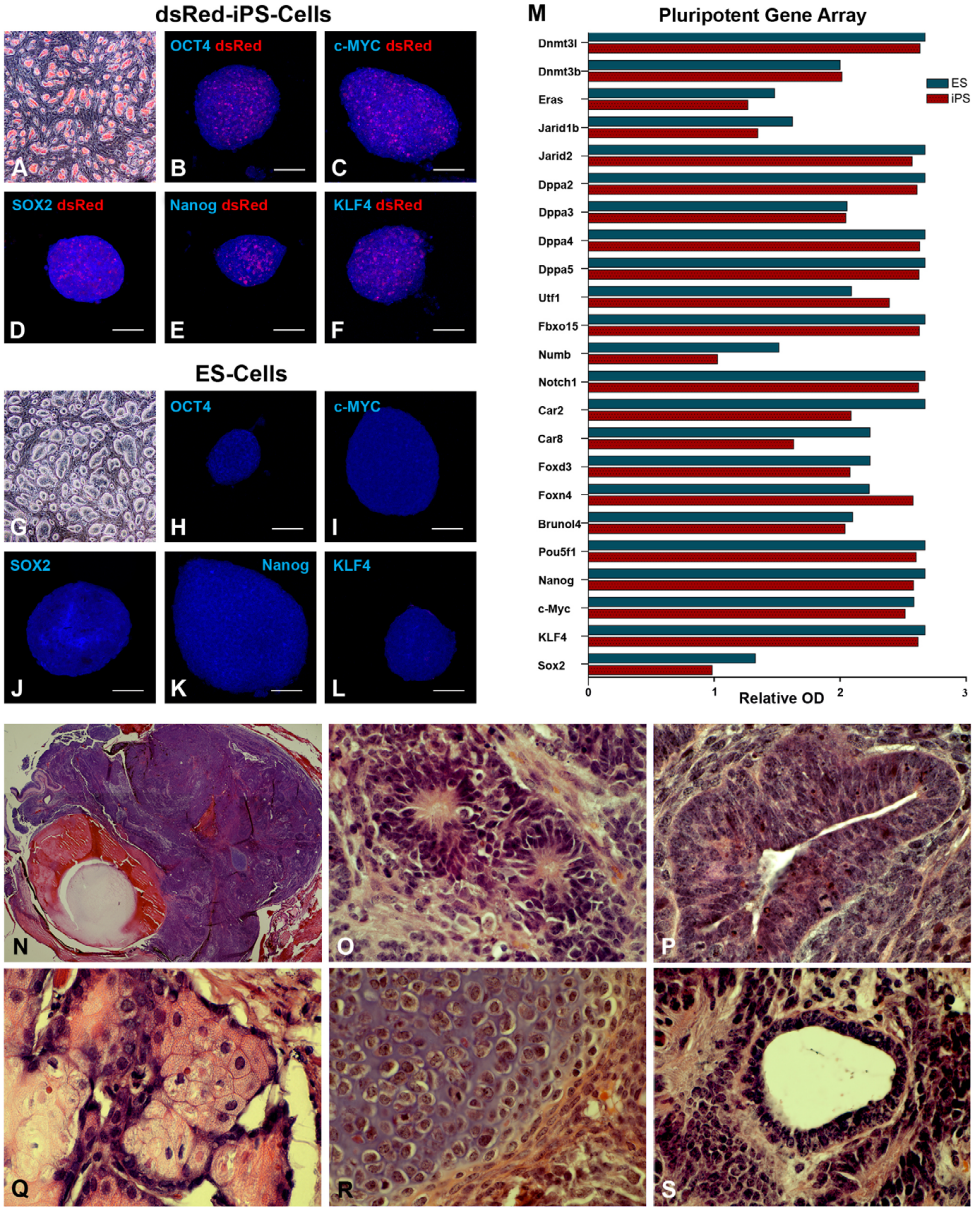
Characterization of adult mouse dsRed-iPS cells. **A–L:** Microscopic/Immunocytochemical analysis comparing ES and dsRed-iPS cell morphology (**A, G**) and expression of the pluripotency markers Oct4 (**B, H**), c-MYC (**C, I**), Sox2 (**D, J**), Nanog (**E, K**) and Klf4 (**F, L**). **M:** Focused microarray analysis performed on RNA isolated from both ES and dsRed-iPS cells for the expression of genes known to be associated with pluripotency. **N–S:** Histological analysis of dsRed-iPS cell generated teratomas (N) for production of cells/tissues specific to ectodermal **(O&P**) mesodermal (**Q&R**) and endodermal (**S**) germ layers. Scale Bar = 100 µm.

Forced expression of the transcription factors Oct4, Sox2, Klf4, and c-MYC induced formation of cell colonies that were indistinguishable from those of control mouse ES cells (Fig. 1 A, G ). Immunocytochemical analysis revealed that dsRed-iPS cell colonies expressed levels of the markers Oct4 (B), c-MYC (C), Sox2 (D), Nanog (E) and Klf4 (F) that were comparable to mouse ES cells (G, H, I, J&K). Similarly, a focused microarray analysis showed that 23 genes indicative of pluripotency were similarly expressed between mouse ES and dsRed-iPS cells (Fig. 1M). These results demonstrate that the iPS cells generated in this study are similar to established ES cell colonies.

To test for pluripotency, 2.5×10^6^ undifferentiated dsRed-iPS cells were injected into the eye of SCID mice. At 21 days post-transplantation, histological analysis revealed teratomas (Fig. 1N) containing tissue specific to each of the three embryonic germ layers (Fig 1O, P neural rosettes, neuroepithelia—ectoderm; Q, R adipocytes, chondrocytes—mesoderm; S glandular epithelium—endoderm). Similarly, immunocytochemical staining of the teratomas revealed GFAP-positive neural rosettes (Fig. S1A, ectoderm), βIII tubulin-positive neural tissue (Fig. S1B, ectoderm) and α-smooth muscle actin-positive arterial structures (Fig. S1C, mesoderm). Collectively, these findings demonstrate that we have successfully produced a reprogrammed adult mouse dsRed-iPS cell line that possesses the ability to produce cell types of all three germ layers, similar to embryonic stem cells.

**Figure 2:**
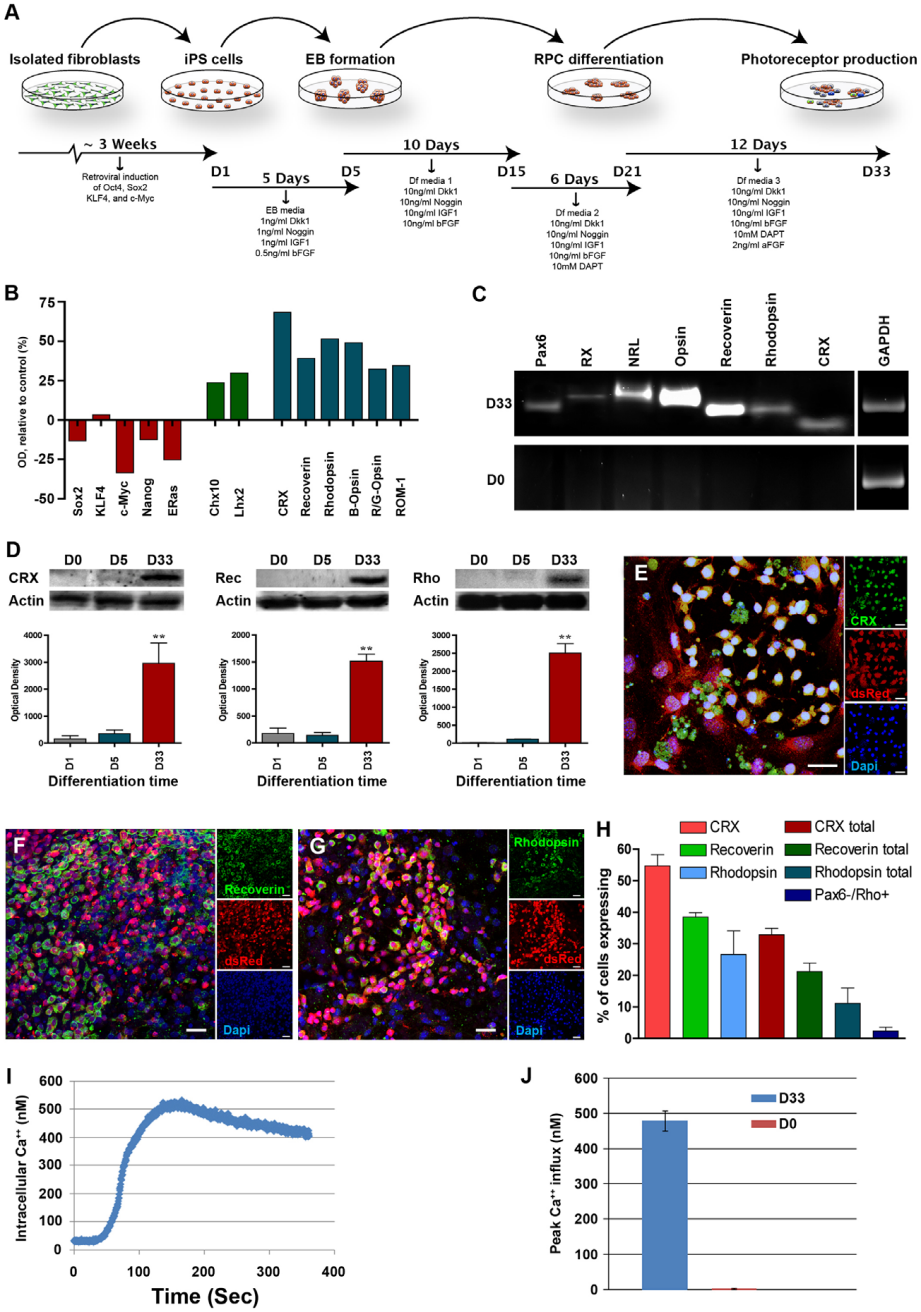
Differentiation of dsRed-iPS cells into retinal photoreceptors using defined culture conditions. **A:** Schematic diagram illustrating the differentiation paradigm utilized in this study. **B:** Focused microarray analysis of undifferentiated (D0) and differentiated (D33) dsRed-iPS cells for pluripotency (Sox2, Klf4, c-MYC, Nanog and Eras), retinal progenitor (Chx10 and Lhx2) and retinal photoreceptor (CRX, recoverin, rhodopsin, blue-opsin, red/green-opsin and ROM-1) gene expression. **C:** RT-PCR analysis of undifferentiated (D0) and differentiated (D33) dsRed-iPS cells for expression of the retinal specification/photoreceptor genes Pax6, RX, NRL, cone-opsin, rhodopsin, recoverin and CRX (GAPDH was including as an internal control). **D:** Western blot analysis of D0 undifferentiated, D5 embryoid bodies and D33 differentiated dsRed-iPS cells for expression of the retinal photoreceptor markers CRX, recoverin and rhodopsin. **E–G:** Immunocytochemical analysis of photoreceptor marker (CRX, recoverin and rhodopsin) expression. **H:** Average number of cells expressing the photoreceptor markers CRX, recoverin and rhodopsin at D33 post-differentiation. **I–J:** Intracellular Ca^2+^ levels as a function of time (**I**) and peak (**J**) intracellular Ca^2+^ concentrations for D0 undifferentiated and D33 differentiated iPS cells in response to 1 nM glutamate stimulation. Scale bar = 25µm. *p<0.05, **p<0.001. ***p<0.0001.

To produce retinal neurons for subretinal transplantation, a stepwise differentiation protocol was developed. This protocol combines different aspects of previously published ES and iPS cell differentiation paradigms [7], [8], [9], [24], [25] so as to maximize the percentage of photoreceptor cells produced for transplantation (Fig. 2A). This protocol takes into account the role of bone morphogenic protein (BMP) and Wnt signaling pathway inhibition in neuroectodermal development [26], [27], [28], as well as the role of IGF-1 in anterior neural/eye field development [29] and Notch pathway inhibition in photoreceptor development [30]. This paradigm takes into account basic knowledge established for eyefield and retinal development, using extrinsic chemical signaling pathways to manipulate cell fate.

To determine whether the above protocol was effective at inducing retinal cell differentiation, experiments using total RNA isolated from D0 undifferentiated and D33 differentiated dsRed-iPS cells were performed. As shown in Figure 2B, the pluripotency genes Sox2, c-MYC, Nanog, and Eras decreased (red bars), while the retinal progenitor cell genes Chx10 and Lhx2 (green bars) and the photoreceptor cell genes CRX, recoverin, rhodopsin, blue-opsin, red/green-opsin and ROM-1 (blue bars) increased in D33 differentiated cells in comparison to D0 undifferentiated controls. Similarly, increased expression of the retinal cell markers Pax6 and RX and the retinal photoreceptor markers NRL, cone-opsin, recoverin, rhodopsin and CRX was detected by RT-PCR (Fig. 2C, gene specific primer sequences can be found in Table S1). These data indicate that following a 33 day differentiation protocol leads to production retinal neurons from iPS cells.

To confirm the presence of retinal photoreceptor marker expression, lysates from D0 undifferentiated cells, D5 embryoid bodies and D33 differentiated cells were analyzed via western blotting. Blots were probed for expression of the pan photoreceptor marker CRX and the rod photoreceptor markers recoverin and rhodopsin (Fig. 2D). As expected, neither CRX, recoverin nor rhodopsin could be detected in D0 undifferentiated cultures (Fig. 2D). Although a slight increase in CRX expression was detected in D5 embryoid bodies, neither recoverin nor rhodopsin expression could be detected at this time point (Fig. 2D). In comparison to cells at either D0 or D5, D33 differentiated cells showed significantly elevated expression of CRX, recoverin and rhodopsin (Fig. 2D), further supporting the fact that retinal photoreceptors were being formed following a 33 day differentiation paradigm.

Microscopically, at D33 clonal areas of differentiation were evident, i.e. clusters of differentiated and undifferentiated cells could be identified. Within the differentiated cell clusters, immunocytochemical staining revealed that approximately 55% of the cells expressed CRX (Fig. 2E, H), 40% of the cells expressed recoverin (Fig. 2F, H), and 28% of the cells expressed rhodopsin (Fig. 2G, H). To provide a representative depiction of the entire heterogenous cell population at the end of the differentiation protocol, all cells within the culture system, differentiated and non-differentiated alike, were counted. As shown in figure 2, CRX, recoverin, and rhodopsin were expressed by approximately 33%, 22%, and 12% of the cells respectively (H, total bars). To determine the percentage of mature photoreceptor precursor cells present at day 33 post-differentiation, immunocytochemical analysis targeted against Pax6 and rhodopsin was performed (i.e. Pax6 is expressed in developing retinal progenitor cells and turned off in mature photoreceptors). As indicated by the PAx6-/Rho+ bar in figure 2H, approximately 1–3% of the cells within the heterogeneous cultures at day 33 post-differentiation were found to be mature PAx6-negative rhodopsin-positive photoreceptor precursors. In addition to photoreceptors, other retinal cell types such as NF200-expressing retinal ganglion cells (Fig. S2A), GS-expressing glial cells (Fig. S2B) and ZO-1-expressing retinal-pigmented epithelial cells (Fig. S2C) were also identified. These data indicate that retinal cell types of several lineages could be produced from iPSCs.

Under normal conditions, retinal progenitor and photoreceptor precursor cells have been shown to express functional ionotropic glutamate receptors and respond to neurotransmitter stimulation with Ca^2+^ influx [31]. To determine whether D33 differentiated dsRed-iPSCs had progressed along a retinal progenitor/photoreceptor precursor lineage, calcium-imaging experiments were performed. D33 differentiated iPSC derived retinal progenitor cells responded to 1 nM glutamate stimulation with an increase in intracellular Ca^2+^ levels over time (Fig. 2I). Compared to D0 undifferentiated controls, a significant increase in peak intracellular Ca^2+^ concentration was observed in D33 differentiated populations (Fig. 2J). This suggests that iPSC-derived retinal progenitor cells exhibit functional membrane properties consistent with the desired phenotype.

Despite the ability to efficiently generate photoreceptor cells using the protocol described above, complete differentiation of D33 cultures was not achieved. As evident by expression of the pluripotency marker SSEA1, approximately 30% of the cells at D33 remained undifferentiated (Fig. S3A, C). These cells were predominantly located at the perimeter of originally plated embryoid bodies (Fig. S3A). The early pan-retinal marker Pax6, although significantly down regulated post-differentiation, could be detected in as much as 65% of the cells at D33, in contrast to the SSEA1-positive profile, these cells were not restricted to the perimeter of embryoid bodies (Fig. S3B, C). These data indicate that although substantial differentiation was achieved, it remained incomplete.

**Figure 3:**
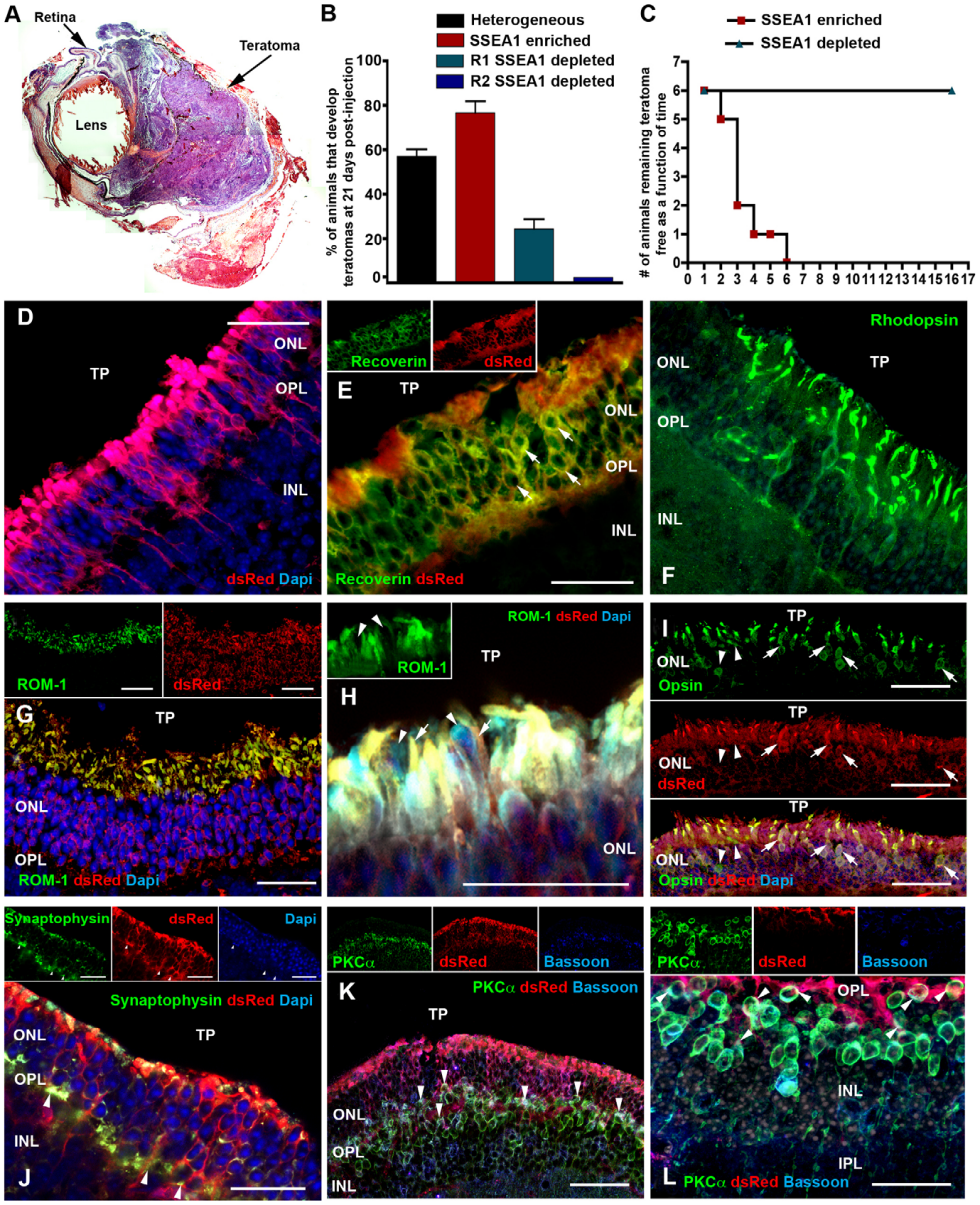
Transplantation of SSEA1- dsRed-iPS derived photoreceptor precursor cells induces retinal outer nuclear layer repopulation. **A:** Histological staining of a teratoma containing Rho-/- eye at 21 days post-injection of a heterogeneous population of SSEA1-containing D33 differentiated cells. **B:** Percentage of animals developing teratomas after receiving either heterogeneous undepleted SSEA1-containing (n = 5), SSEA1-enriched (n = 5), one round of SSEA1-depleted (n = 10) or two round of SSEA1-depleted cell transplants at 21 days post injection. **C:** Number of animals to and time taken for the development of teratomas in animals receiving either SSEA1-enriched or SSEA1-depleted cell transplants over a 16 week post-op period. **D–L:** Immunocytochemical analysis performed on rho-/- retinal degenerative eyes 21 days after receiving subretinal injections of SSEA1-depleted cells targeted against expression of the photoreceptor markers recoverin (E), rhodopsin (F), ROM-1 (G&H) and opsin (I), the synapse markers synaptophysin (J) and bassoon (K&L), the bipolar cell marker PKCα (K&L) and the iPS cell marker dsRed (D–J). TP = transplant site. Scale bar = 50 µm.

As transplantation of the above-mentioned heterogeneous population of cells induced teratoma formation at 21 days post-transplantation (Fig. 3A&Fig. S4), removal of the undifferentiated cells from the cultures prior to delivery was required. To do so, magnetic bead depletion of the SSEA1-expressing cell population was performed. To achieve maximal removal of SSEA1-expressing cells, the depletion protocol as described in Figure S3 was performed twice in series (D). Following depletion, SSEA1-negative cultures were approximately 98% free from residual SSEA1-positive cell contamination (Fig. S3E, G) and nearly 70% of SSEA1-enriched cultures were composed of SSEA1-expressing cells (Fig. S3F, G).

Unlike the results obtained following transplantation of either heterogeneous or SSEA1-enriched cell populations, in which rapid teratoma formation was observed in approximately 60 and 80 percent of recipient animals by 21 days post-injection respectively (Fig. 3B), approximately 20 percent of the recipient animals that received cells that underwent a single round of SSEA1-expressing cell depletion developed tumors (Fig 3B). Importantly, following a second consecutive round of SSEA1-expressing cell depletion, at 21 days post-injection none of the retinal degenerative recipient animals developed tumors (Fig. 3B). However, when staining for the proliferation marker Ki67, a small collection of proliferative cells within the vitreal space within one of the animals that had received an SSEA1-depleted injection was observed (Fig. S5A). Therefore, although we did in fact eliminate tumor formation with this protocol, the persistence of proliferating cells suggest further work to avoid potential adverse events following transplantation is needed.

To test further the safety of these cells within the ocular environment, additional transplantation studies were performed in which 12 animals (rho-/-) receiving either SSEA1-enriched (n = 6) or SSEA1-depleted (n = 6) cell populations were allowed to survive for up to 16-weeks post-injection. As expected, only 2 of the 6 animals that received SSEA1-enriched cell injections remained teratoma free and were monitored beyond 3-weeks post-transplantation (1 of the 4 animals in this group developed a tumor and had to be euthanized at 1 week post-injection; Fig 3C). After an additional two weeks, the remaining two animals developed obvious tumors and were euthanized (Fig. 3C). In contrast, none of the 6 animals that received the SSEA1-depleted cell injections developed teratomas by 16 weeks post-transplantation (Fig. 3C). Ki67 positive cells were not detected in any of the animals that received the SSEA1-depleted cell transplants at 16 weeks post-injection (Fig S5B). These results make it clear that tumor formation can be reliably eliminated from long -term transplant recipients.

Immunocytochemical analysis revealed extensive cellular integration and retinal photoreceptor/outer nuclear layer repopulation following subretinal transplantation of D33 differentiated SSEA1-negative cells (Fig. 3D, Fig. S6A–B, and Fig. S6D showing an absence of positive dsRed staining in the contralateral non-injected eye, i.e. as differentiation leads to a downregulation of dsRed transgene expression (see Fig 2E–G,), to allow us to confidently detect all of the donor cells post-transplantation, unless otherwise stated, counterstaining with a dsRed antibody was performed). In the best examples, approximately 6.4% of the cells transplanted integrate within the ONL at 3–4 weeks post-transplantation (i.e.∼16,000 of 250,000 cells, unlike the SSEA1-enriched population, residual unintegrated SSEA1-negative cells could not be identified. The most probable explanation for this result is cell death due to lack of cellular support post-transplantation, i.e. we previously found that when differentiated retinal cells are transplanted on a biodegradable polymer based support structure a 9-fold increase in survival and integration could be achieved [32].). Transplanted cells that took up residence within the outer nuclear layer developed the proper photoreceptor morphology (Fig. S6C, single cell located at the peripheral limit of engraftment) and were found to express the rod photoreceptor markers recoverin (Fig. 3E) and rhodopsin (Fig. 3F, as these transplants were performed in rhodopsin null animals (Fig. S6E contralateral eye negative for rhodopsin expression) co-labeling with dsRed was not required) and the rod outer segment marker ROM-1 (Fig. 3G). Under higher magnification it was evident that ROM-1 tightly co-localized with the donor cell reporter gene dsRed (Fig. 3H, arrows). In contrast, host photoreceptors, which lack rod outer segments in the rho-/- model [33], [34], do not express this proposed pattern of ROM-1 staining, especially following degeneration-induced rod photoreceptor denudation [34] (Fig. 3G, arrowheads, Fig. S6D control contralateral eye). As with rod photoreceptor markers, cone-opsin (i.e. co-labeled with antibodies directed against both red/green and blue cone-opsin) was also expressed by a significant population of donor-derived photoreceptor cells (indicated by co-localization of dsRed and cone-opsins (red/green and blue cone-opsin) within both the newly formed cell bodies and cone outer segments; Fig. 3I, arrows and Fig. S7C). Very few of the cone photoreceptor cells identified in these studies were of host rather than donor origin (Fig. 3I and Fig. S7A-C arrowheads), indicating non-dsRed positive cell and outer segments expressing pan cone-opsin (Fig. 3I, Fig. S7C, co-labeled with red/green and blue cone-opsin), blue cone-opsin (Fig. S7A) and red/green cone-opsin (Fig. S7B) respectively. These data demonstrate that engrafted progenitor cells can differentiate into mature cone photoreceptors following transplantation.

To determine whether transplanted iPS cell-derived photoreceptors integrate within the host retinal circuitry, immunocytochemical analysis of synaptic marker expression was performed. Pronounced co-localization of the synaptic marker synaptophysin and the donor cell marker dsRed was observed (Fig. 3J, arrowheads), particularly at the level of the outer plexiform layer. Similarly, co-localization of synapsin and dsRed (Fig. S8A, arrowheads) and VAMP2 and dsRed (Fig. S8B, arrowheads) were identified. To further demonstrate coaptation of dsRed positive iPS cell-derived photoreceptors processes with host bipolar cells post-transplantation, immunocytochemical staining targeted against the synaptic marker bassoon, the bipolar cell marker PKCα and the donor cell marker dsRed were performed. As shown in figure 3, extensive co-localization among all 3 markers, with clear punctate bassoon staining within the OPL, was identified (Fig. 3K&L arrowheads, low and high magnification images respectively). We also demonstrate the differentiation/integration of a modest number of cells into other retinal cell types, i.e. staining for NF200 and GFAP indicative of retinal ganglion cells (Figure S9A) and Müller glia (Figure S9B). These results make it clear that grafted cells form new synapses with the host retina, leading us to investigate the possibility of functional recovery in recipient animals.

**Figure 4:**
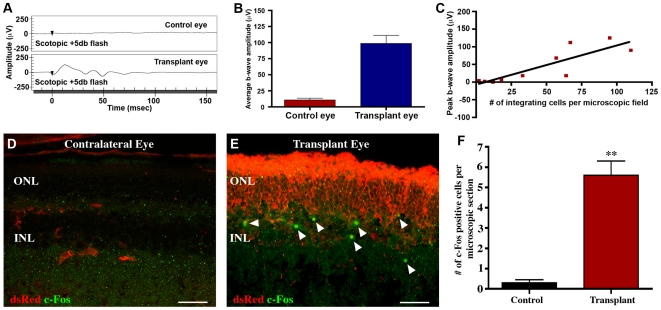
Transplantation of SSEA1-dsRed-iPS derived photoreceptor precursor cells induces increased electroretinal function as determined by ERG and light induced c-Fos expression. **A–C:** Representative ERG (A, 5 db flash under scotopic conditions), average peak b-wave amplitudes for rho-/- mice 21 days after receiving subretinal SSEA1-depleted cell injections (B, n = 6 of 10 animals that received transplants, only animals with recovery in ERG response above baseline were chosen for this analysis, the 4 animals that were not chosen were found to have poor cellular integration due to extensive cell death post- transplantation), and correlation between peak b-wave amplitude and iPS cell derived photoreceptor layer repopulation (C, n = 10). **D–E:** Immunocytochemical analysis targeted against the immediate early gene c-Fos and the iPSC marker dsRed performed on rho-/- transplant and contralateral control mouse eyes at 21 days post-subretinal injection of SSEA1-negative dsRed-iPS derived photoreceptor precursor cells. **F:** Number of cells per microscopic section expressing c-Fos. A significant increase in the number of cells within the retinal inner nuclear layer expressing c-Fos was detected in Rho-/- eyes that had received subretinal injections of SSEA1-negative dsRed-iPSC derived photoreceptor precursor cells as compared to contralateral control eyes following light exposure. Scale bar = 50 µm.

To determine whether integrated iPS-derived photoreceptor cells enable recovery of retinal functional, electroretinographic (ERG) analysis of rho-/- animal eyes, with or without transplants at 28 days post-op, was performed (at the time of recording animals were 8–10 weeks of age). To ensure that changes in visual function post-transplantation were due to the presence of new graft-derived photoreceptor cells and not related to residual host cones, retinal degenerative recipient animals were analyzed pre-injection and chosen based on absence of detectable b-wave amplitude. As shown in Figure 4, a statistically significant increase of approximately 95 µv in b-wave amplitude was observed at 21 days post-subretinal transplant as compared to the contralateral uninjected eye (A, B). Similar findings have been reported for ES cell-derived photoreceptor precursors in CRX-/- mice, whereby an approximate 40–50 µV increase in b-wave amplitude was observed post-transplantation [6]. The difference in recovered b-wave amplitude can potentially be explained by a variety of factors including increased photoreceptor production via the modified differentiation protocol used here, the use of allogeneic as opposed to xenogeneic donor cells, the fact that remaining undifferentiated cells were removed from the D33 differentiated population prior to transplantation and finally the use of a different host/retinal degeneration model systems (i.e. Rho-/- vs. CRX-/-).

To further demonstrate the relationship between cellular integration and recovery of retinal function, a linear regression analysis was performed. As illustrated in Figure 4, a significant positive correlation between peak b-wave amplitude and donor dsRed-positive photoreceptor cell integration was identified (Fig. 3C, r^2^ = 0.7316, n = 10, p≤0.005). These findings suggest that as more cells integrated within the dystrophic retinal architecture, a larger increase in recovery of electro-retinal function was seen. Although these data suggests that transplanted iPSC-RPCs are integrating functionally within the host retinal architecture (i.e. a recordable ERG could only be detected in 8–10 week old mice post-transplantation), we cannot exclude the possibility that transplant induced restoration of residual host cone viability is partially contributing to the increased ERG response.

To further assess retinal function in grafted versus ungrafted eyes, functional anatomy focused on activation of neurons within the retinal inner nuclear layer (INL) was performed. As light exposure, and subsequent photoreceptor activation/phototransduction, is known to induce nuclear c-Fos expression in the inter-neurons of the INL [35], immunocytochemical analysis targeted against the expression of this marker was employed. As compared to control contralateral ungrafted eyes, which do not express functional rhodopsin and in turn lack significant light induced phototransduction (Fig 4D&E), eyes that had received iPSC-derived photoreceptor precursors were found to have significantly increased light induced INL c-Fos expression at 21 days post-transplantation (Fig. 4E arrowheads&F). For instance, an approximate 7-fold increase in the number of INL cells expressing c-Fos was detected (Fig. 4F). In large part, c-Fos expressing cells were located in close proximity to newly engrafted dsRed-iPSC derived photoreceptor processes (Fig. 4E arrowheads).

Collectively, these data suggest that adult dermal fibroblast-derived iPS cells represent a useful source of replacement retinal neurons, particularly photoreceptors, and that this method of treatment is capable of providing at least partial restoration of retinal function. It is worth noting that such an approach in genetic dystrophies such as retinitis pigmentosa will require either gene correction, as employed in an animal model of sickle cell anemia [36], or engraftment with normal allogeneic tissue, as patient specific cells will express the same genetic defect found in host photoreceptors. Moreover, further investigations into large animal models of diseases resembling age-related macular degeneration will be needed if any restorative transplantation strategies are to be employed in this disorder.

These data also demonstrate the potential utility of active removal of residual pluripotent cells prior to transplantation. As embryonic stem cells that have not been subject to manipulation with viral vectors do not appear to possess the same propensity toward tumor formation post-transplantation [6], [37], [38], alternate means of adult dermal cell reprogramming, which do not utilize genome-incorporating viruses, would be advantageous. It is reasonable to expect that sustained over-expression of the transgenes used to induce pluripotency might interfere with terminal differentiation and the data presented in Figure 2B, showing maintenance of Klf4 gene expression in heterogenous unselected cultures following differentiation, would support this. Efficient removal of undifferentiated SSEA1-expressing cells mitigates the risk of teratoma formation, thereby allowing for improved experimental outcome including orthotopic replacement of photoreceptors and partial restoration of electroretinal function.

## Materials and Methods

### Ethics statement

All experiments were conducted with the approval of the Schepens Eye Research Institute Animal Care and Use Committee (Animal welfare assurance # A3177-01, ACUC approval # S-170-0710) and the ARVO Statement for the Use of Animals in Ophthalmic and Vision Research.

### Animals

Adult 4–6 week old dsRed-positive C57Bl6 mice (Jackson Laboratory, Bar Harbor, ME) were used as fibroblast donors; adult 4–6 week old rhodopsin-null mice that lack rod outer segments and do not form functional rod photoreceptors [33] (rho-/-, Peter Humphries, Trinity College, Dublin) were used as retinal degenerative transplant recipients. Severe combined immunodeficient mice (SCID, Jackson Laboratory, Bar Harbor, ME) were used for assessment of teratoma formation.

### iPS cell differentiation

To maintain pluripotency, adult dsRed-iPS cells were cultured on inactive mouse embryonic fibroblasts in LIF containing pluripotency media. To begin differentiation, iPS cells are removed from the culture substrate via incubation in a 1 mg/ml type I collagenase (Sigma-Aldrich) solution, resuspended in embryoid body media (DMEM F-12 media (Gibco) containing 10% knockout serum replacement (Gibco) 2% B27 supplement (Gibco) 1% N2 supplement (Gibco), 1% L-glutamine (Gibco), 1% 100x NEAA (Gibco), 1% penicillin/streptomycin (Gibco), 0.2% Fungizone (Gibco), 1 ng/ml noggin (R&D Systems, Minneapolis, MN), 1 ng/ml Dkk-1 (R&D Systems), 1 ng/ml IGF-1 (R&D Systems) and 0.5 ng/ml bFGF (R&D Systems)), and plated at a density of ∼50 cell clumps/cm^2^ in ultra low cluster plates (Corning, Lowell, MA). Cell clumps are cultured for 5 days as indicated above, after which the embryoid bodies are removed, washed and plated at a density of 25–30/cm^2^ in fresh differentiation media 1 (DMEM F-12 media (Gibco), 2% B27 supplement (Gibco) 1% N2 supplement (Gibco), 1% L-glutamine (Gibco), 1% 100x NEAA (Gibco) 10 ng/ml noggin (R&D Systems), 10 ng/ml Dkk-1 (R&D Systems), 10 ng/ml IGF-1 (R&D Systems) and 1 ng/ml bFGF (R&D Systems)) in 6-well culture plates coated with poly-D-lysine (BD Bioscience, San Jose, CA, 10 mg/ml), collagen (BD Bioscience, 25 µg/ml), laminin (Gibco, 50 µg/ml) and fibronectin (Sigma-Aldrich, 100 µg/ml). Cultures are fed every other day for 10 days with differentiation media 1, then every other day for an additional 6 days with differentiation media 2 (differentiation media 1+10 uM of the Notch signaling inhibitor, DAPT (Calbiochem, Gibbstown, NJ), followed every other day for an additional 12 days with differentiation media 3 (differentiation media 2+2 ng/ml of aFGF (R&D Systems)).

### ERG

8–10 week old rhodopsin null transplant recipient mice were dark-adapted for 12 hrs prior to testing, anesthetized, had their pupils dilated by topical application of tropicamide (Akorn, Lake Forest, IL) and placed on a heated recording stage maintained at 37°C. Contact lens electrodes were placed directly onto the corneal surface of eyes precoated with a 2.5% hydroxypropylmethylcellulose solution (Gonak, Akorn), while a copper reference electrode was placed beneath the scalp and another ground was inserted beneath the tail skin. Responses to 5 test flashes were recorded for each mouse and all ERGs were carried out under scotopic conditions using a standard 5 db flash. ERG signals were amplified 10,000x, filtered between 1 Hz to 3 kHz, and sampled at 5 kHz. All recordings were performed under infrared light and data was analyzed using EMwin software (LKC Technologies, Inc., Gaithersburg, MD). Data present in Figure 3 were taken from responsive animals at 21 days post-transplantation (M).

### Functional anatomy

To further assess retinal function post-transplantation, analysis of c-Fos expression was performed. Rho-/- null transplant animals housed in a 12 hr dark/light cycle were sacrificed at approximately 1.5 hr after the lights had been turned on in their housing environment (∼280 mW/cm^2^). Animals were subsequently sacrificed and enucleated. The eyes were fixed in 4%PFA, cryosectioned and immunostained with a primary antibody targeted against c-Fos (Calbiochem, Gibbstown, NJ) and detected using a cy2 conjugated secondary antibody (Jackson, West Grove, PA).

For further details pertaining to Retroviral production and iPS cell generation, Focused Microarray Analysis, Subretinal Transplantation, SSEA1 Cell depletion, Calcium imaging, Immunostaining, Immunoblotting, RNA isolation and RT-PCR, Cell Counting, and Statistical Analysis see Methods S1.

## Supporting Information

Figure S1 **Analysis of dsRed-iPS cell pluripotency. A–C:** Immunocytochemical analysis performed on dsRed-iPS cell derived teratomas targeted against the ectodermal markers GFAP (A: glia) and βIII tubulin (B, neural), and the mesodermal marker α-SMA (C: vascular). Expression of ectodermal (GFAP and NF200) and mesodermal (α-SMA) markers within iPS cell derived teratomas indicate that the parent iPS cells are pluripotent.
(TIF)

Figure S2 **Identification of dsRed-iPS cell derived retinal cells. A–C:** Immunocytochemical analysis performed on dsRed-iPS cell cultures at D33 post-differentiation directed against the ganglion cell marker NF200 (A), the glial cell marker GS (B), and the RPE cell/tight junction marker ZO-1 (D). Scale bar = 25 µm.
(TIF)

Figure S3 **Depletion of D33 undifferentiated SSEA1-positive dsRed-iPS cells prior to transplantation prevents teratoma formation. A–B:** Immunocytochemical analysis of SSEA1 and Pax6 expression in D33 cultures post-differentiation. **C:** Percentage of cells expressing SSEA1 and Pax6 in D33 cultures post-differentiated. **D:** Schematic diagram illustrating the procedures used for depletion of remaining SSEA1-positive undifferentiated cells from D33 cultures post-differentiation. **E–F:** Immunocytochemical analysis of SSEA1 expression in SSEA1 cell-depleted and -enriched D33 post-differentiation cultures. **G:** percent of SSEA1-negative cells in SSEA1-depleted cultures and SSEA1-positive cells in SSEA1-enriched cultures following successive rounds of depletion/isolation. Scale bar = 50 µm.
(TIF)

Figure S4 **Transplantation of a heterogeneous population of D33 differentiated dsRed-iPS cells induces teratoma formation. A–B:** Immunocytochemical analysis of recoverin (**A**) and rhodopsin (**B**) expression post-subretinal transplantation of heterogeneous D33 differentiated cells. Transplantation of a heterogeneous population of undepleted cells (i.e. SSEA1 positive population included) isolated at D33 post-differentiation induced either teratomas or at the very least collections of cells suggestive of incipient tumors at 21 days post-transplantation. Cells contained within these masses were found to express both recoverin (A) and rhodopsin (B).
(TIF)

Figure S5 **Proliferative cells identified at 21-days post-transplantation are absent at 16-weeks post-transplantation. A–B:** Immunocytochemical analysis performed on rho-/- recipient mouse eyes at 21-days and 16-weeks post-intravitreal injection of SSEA1-negative dsRed-iPS cells targeted against the cell cycle marker Ki67. Scale bar = 50 µm.
(TIF)

Figure S6 **Transplantation of SSEA1- dsRed-iPS derived photoreceptor precursor cells induces extensive cellular integration and outernuclear layer repopulation. A–C:** Immunocytochemical analysis performed on rho-/- recipient mouse eyes at 21 days post-subretinal injection of SSEA1-negative dsRed-iPS cells targeted against the host donor cell marker dsRed. A–B: Low magnification images used to show the extent of cellular integration post-transplantation. C: High magnification image taken at the outer limit of cellular migration in figure B where sparse cellular integration was observed. This image was taken in an attempt to show detailed donor cell morphology. As shown in these images extensive cellular integration and retinal ONL repopulation was identified across a wide area of the host retina at 3 weeks post-transplantation (A–B). Cells that integrate within the retinal degenerative environment adopt a photoreceptor morphology represented by a single cell body with and outer segment extended toward the RPE and an inner process ending with a synaptic pedicle that extends into the host plexiform layer (C). **D–E:** Immunocytochemical analysis performed on control rho-/- un-injected contralateral mouse eyes at 21 days post-op against dsRed, ROM1 and recoverin (D) or rhodopsin (E). Contralateral 6–8 week old rhodopsin null mouse eyes do not express ROM1, dsRed or rhodopsin, indicating that rod photoreceptors detected in Rho-/- eye at 21-days post-subretinal injection are iPSC transplant derived.
(TIF)

Figure S7 **Generation of cone photoreceptors following transplantation of SSEA1- dsRed-iPS derived photoreceptor precursor cells. A–C:** Immunocytochemical analysis performed on rho-/- recipient mouse eyes at 21 days post-subretinal injection of SSEA1-negative dsRed-iPS cells targeted against the blue cone photoreceptor marker blue-opsin (A), the red/green cone photoreceptor marker red/green-opsin (B) and the pan cone photoreceptor marker pan-cone-opsin (C).
(TIF)

Figure S8 **Synaptic integration of dsRed-iPS cell derived photoreceptor precursor cells following subretinal transplantation. A–C:** Immunocytochemical analysis performed on rho-/- recipient mouse eyes at 21 days post-subretinal injection of SSEA1-negative dsRed-iPS cells targeted against the synaptic markers synapsin (A) and Vamp-2 (B). Scale bar = 10 µm. Transplanted dsRed-expressing iPS cell derived photoreceptor precursors that integrate into the outer nuclear layer of retinal degenerative mice following subretinal injection form synaptic connections at the level of the outer plexiform layer within the host retina.
(TIF)

Figure S9 **Integration of cell types other then retinal photoreceptors at 21-days post-injection. A-C:** Immunocytochemical analysis performed on rho-/- recipient mouse eyes at 21 days post-injection of SSEA1-negative dsRed-iPS cells targeted against the retinal ganglion cell marker NF200 (A) and the glial cell marker GFAP (B). Scale bar = 10 µm. In addition to photoreceptors, transplanted dsRed-expressing iPS cells also gave rise to NF200 expressing retinal ganglion and GFAP-expressing glial cells following ocular injection. Importantly, both cell types took up residence within the appropriate retinal layer and appeared to develop morphologically into the correct cell types.
(TIF)

Table S1 **Gene specific primer sequences used for RT-PCR.**
(DOC)

Methods S1 **Retroviral production and iPS cell generation.**
(DOC)
