# Correction: Transplantation of Adult Mouse iPS Cell-Derived Photoreceptor Precursors Restores Retinal Structure and Function in Degenerative Mice

**DOI:** 10.1371/journal.pone.0125947

**Published:** 2015-05-07

**Authors:** Budd A. Tucker, In-Hyun Park, Sara D. Qi, Henry J. Klassen, Caihui Jiang, Jing Yao, Stephen Redenti, George Q. Daley, Michael J. Young

The affiliation for the seventh author is incorrect. Stephen Redenti is not affiliated with #1 but with #2 Department of Ophthalmology, Schepens Eye Research Institute, Harvard Medical School, Boston, Massachusetts, United States of America. Dr. Redenti’s current address is: Biochemistry Doctoral Program, The Graduate School and University Center, City University of New York, Department of Biological Sciences, Lehman College, City University of New York, Bronx, New York, United States of America.
