# Supplementary figures and images for: Transplantation of Adult Mouse iPS Cell-Derived Photoreceptor Precursors Restores Retinal Structure and Function in Degenerative Mice
Source: PLoS One. 2011 Apr 29;6(4):e18992. doi: 10.1371/journal.pone.0018992 (PMC3084746; doi:10.1371/journal.pone.0018992)

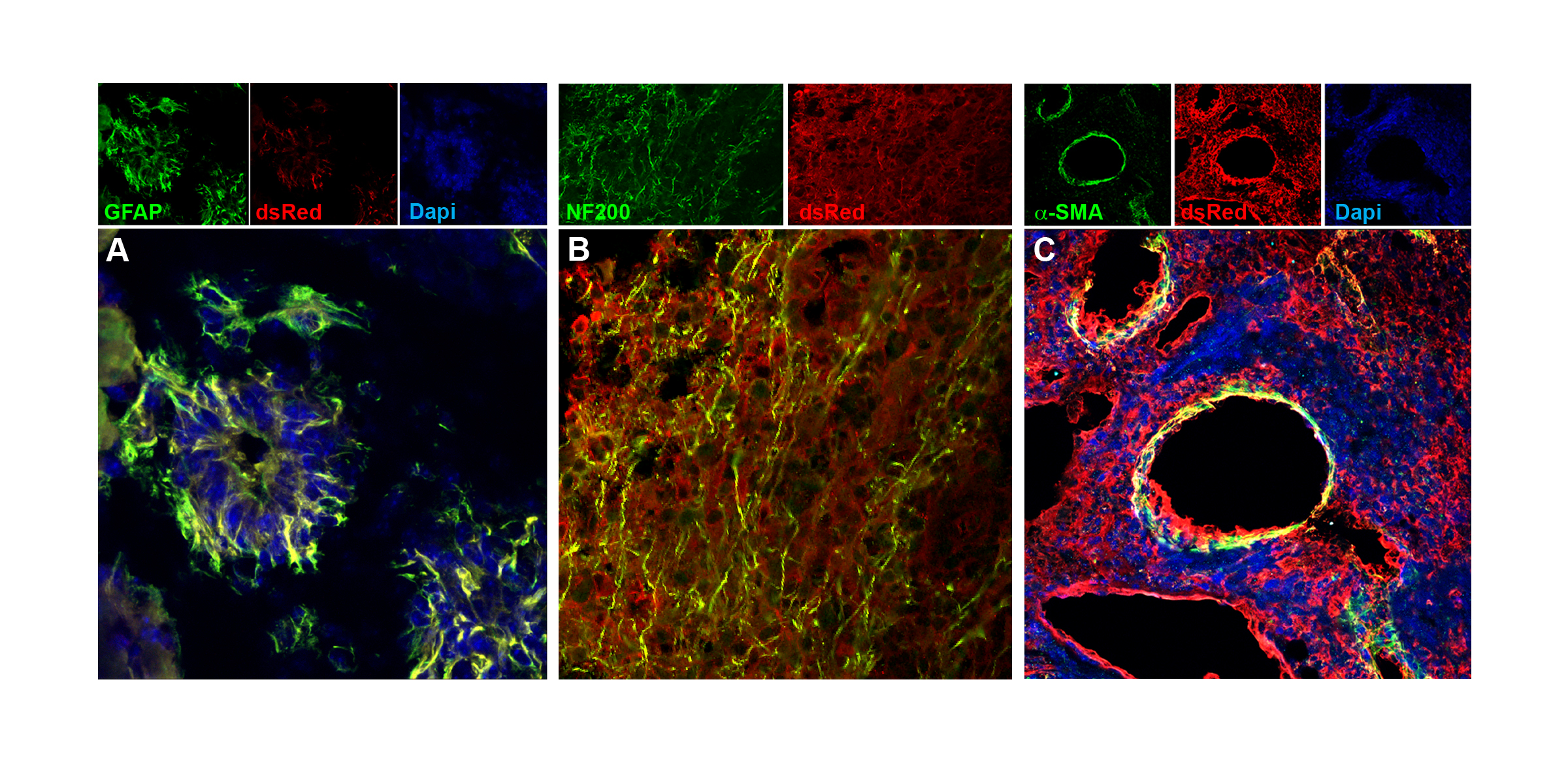

Supplement: Figure S1 — Analysis of dsRed-iPS cell pluripotency. A–C: Immunocytochemical analysis performed on dsRed-iPS cell derived teratomas targeted against the ectodermal markers GFAP (A: glia) and βIII tubulin (B, neural), and the mesodermal marker α-SMA (C: vascular). Expression of ectodermal (GFAP and NF200) and mesodermal (α-SMA) markers within iPS cell derived teratomas indicate that the parent iPS cells are pluripotent. (TIF) [file pone.0018992.s001.tif]

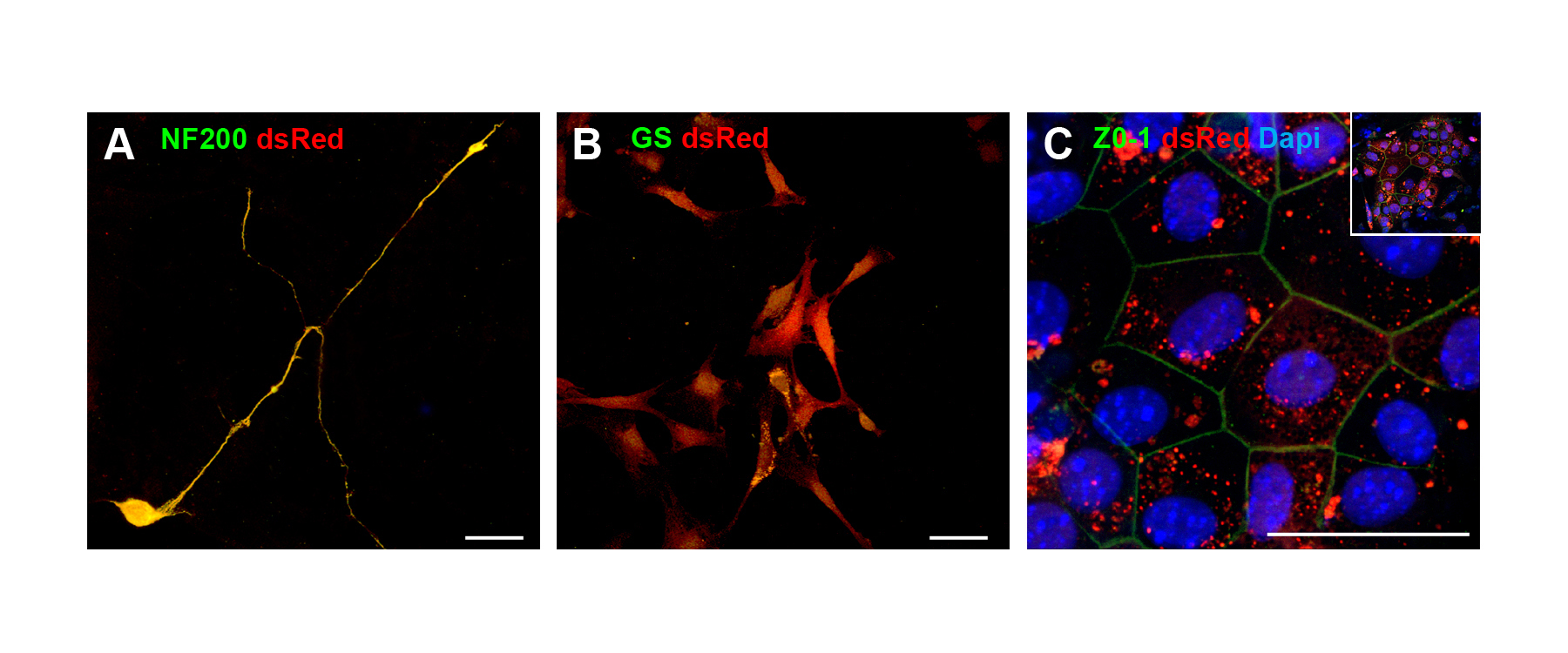

Supplement: Figure S2 — Identification of dsRed-iPS cell derived retinal cells. A–C: Immunocytochemical analysis performed on dsRed-iPS cell cultures at D33 post-differentiation directed against the ganglion cell marker NF200 (A), the glial cell marker GS (B), and the RPE cell/tight junction marker ZO-1 (D). Scale bar = 25 µm. (TIF) [file pone.0018992.s002.tif]

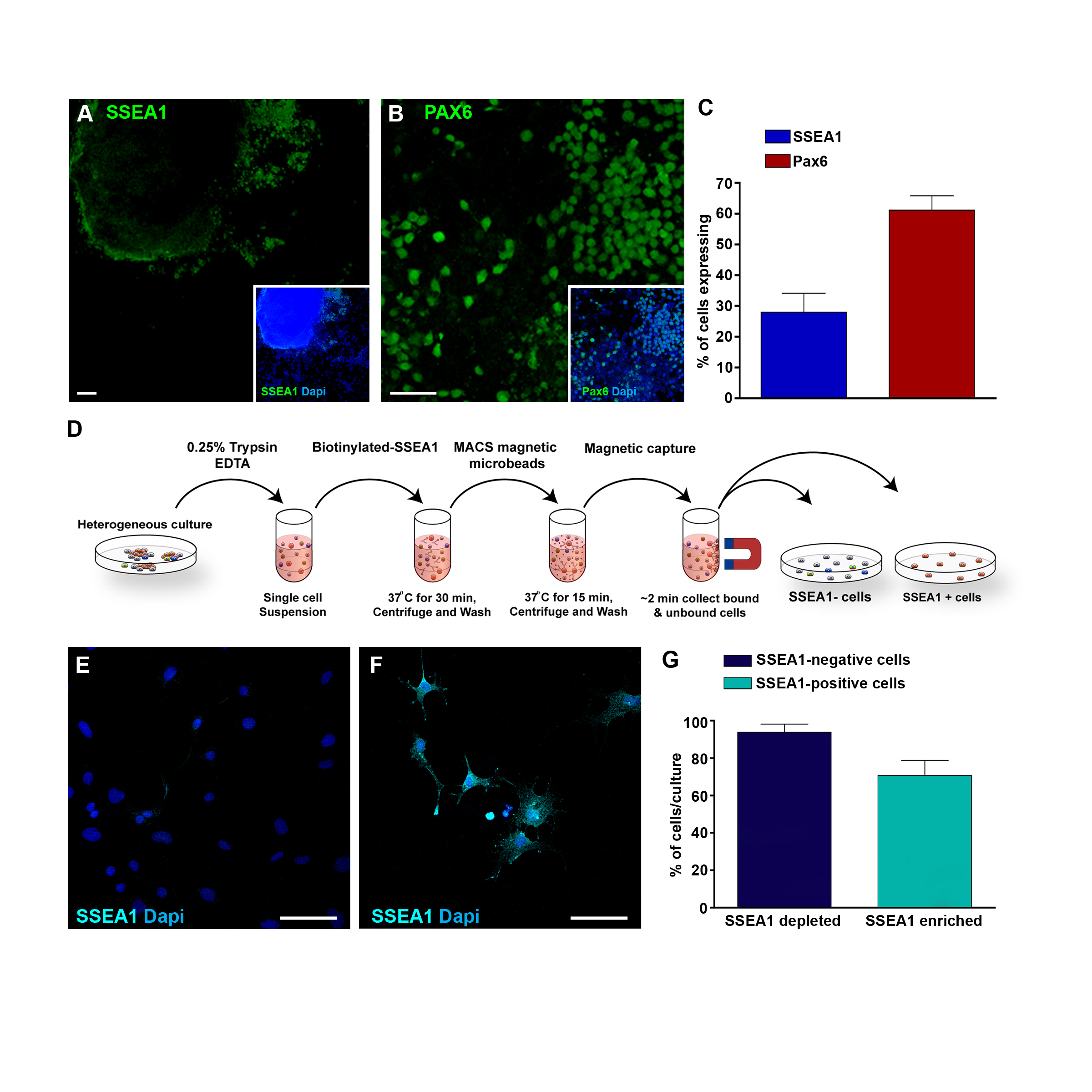

Supplement: Figure S3 — Depletion of D33 undifferentiated SSEA1-positive dsRed-iPS cells prior to transplantation prevents teratoma formation. A–B: Immunocytochemical analysis of SSEA1 and Pax6 expression in D33 cultures post-differentiation. C: Percentage of cells expressing SSEA1 and Pax6 in D33 cultures post-differentiated. D: Schematic diagram illustrating the procedures used for depletion of remaining SSEA1-positive undifferentiated cells from D33 cultures post-differentiation. E–F: Immunocytochemical analysis of SSEA1 expression in SSEA1 cell-depleted and -enriched D33 post-differentiation cultures. G: percent of SSEA1-negative cells in SSEA1-depleted cultures and SSEA1-positive cells in SSEA1-enriched cultures following successive rounds of depletion/isolation. Scale bar = 50 µm. (TIF) [file pone.0018992.s003.tif]

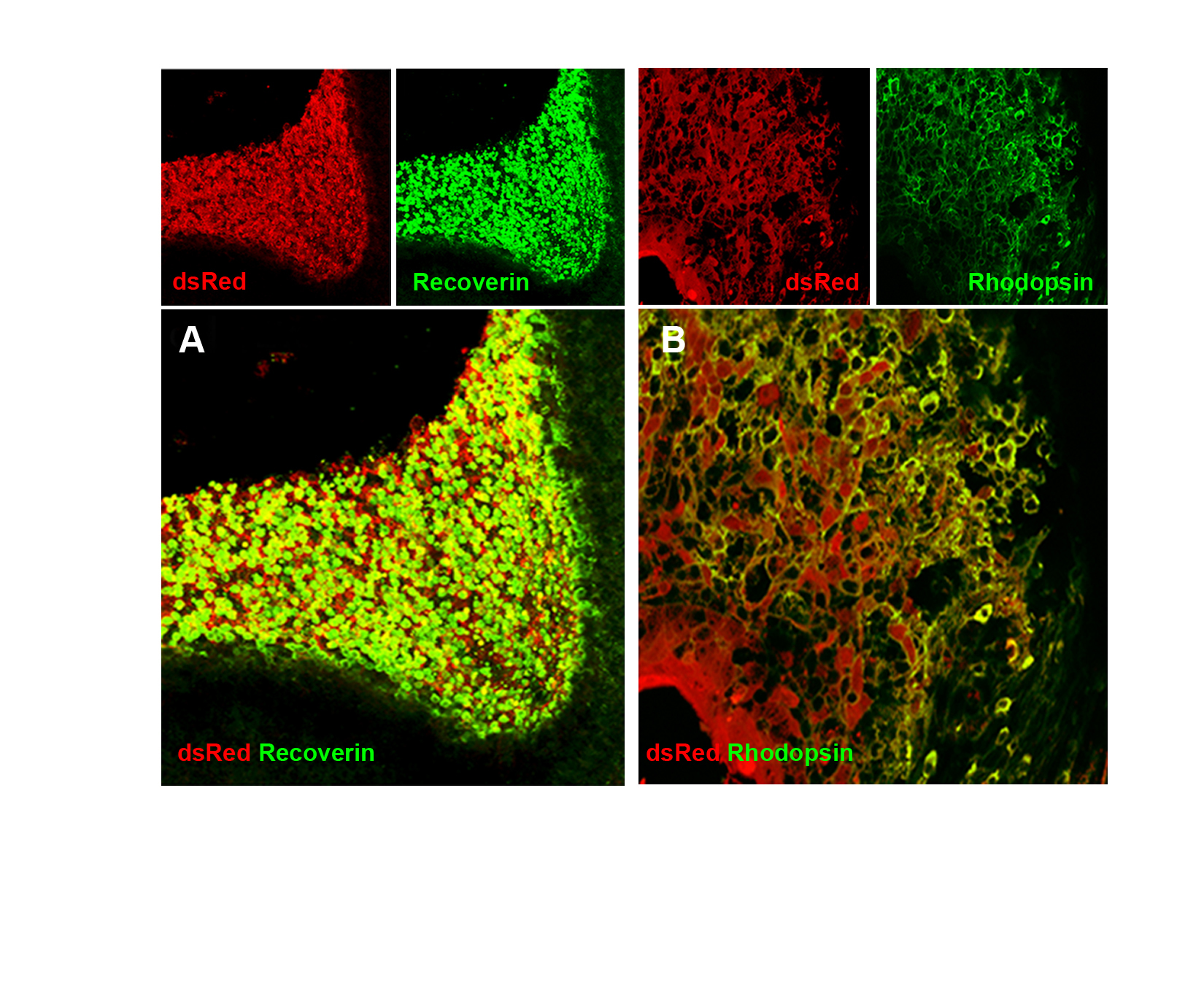

Supplement: Figure S4 — Transplantation of a heterogeneous population of D33 differentiated dsRed-iPS cells induces teratoma formation. A–B: Immunocytochemical analysis of recoverin (A) and rhodopsin (B) expression post-subretinal transplantation of heterogeneous D33 differentiated cells. Transplantation of a heterogeneous population of undepleted cells (i.e. SSEA1 positive population included) isolated at D33 post-differentiation induced either teratomas or at the very least collections of cells suggestive of incipient tumors at 21 days post-transplantation. Cells contained within these masses were found to express both recoverin (A) and rhodopsin (B). (TIF) [file pone.0018992.s004.tif]

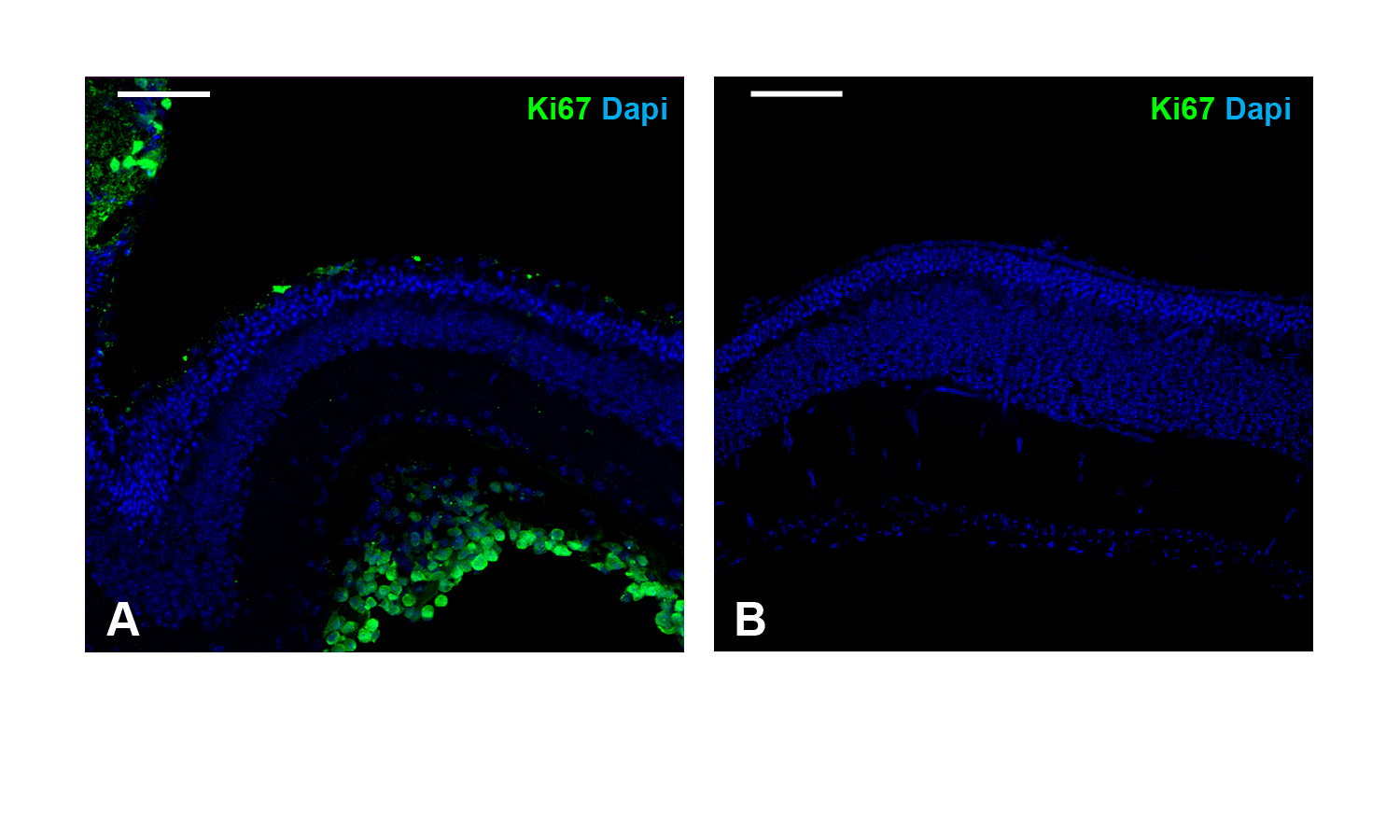

Supplement: Figure S5 — Proliferative cells identified at 21-days post-transplantation are absent at 16-weeks post-transplantation. A–B: Immunocytochemical analysis performed on rho-/- recipient mouse eyes at 21-days and 16-weeks post-intravitreal injection of SSEA1-negative dsRed-iPS cells targeted against the cell cycle marker Ki67. Scale bar = 50 µm. (TIF) [file pone.0018992.s005.tif]

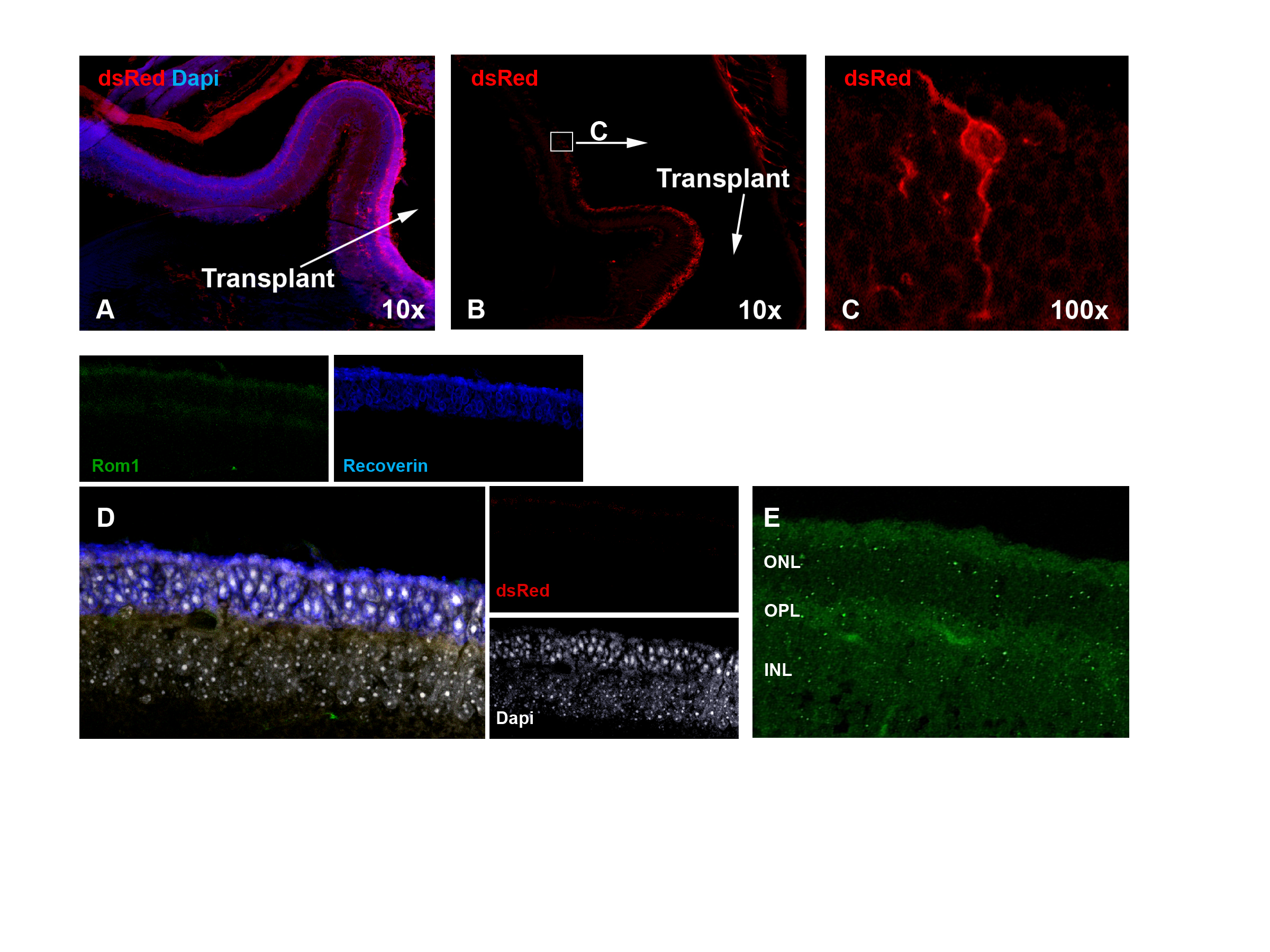

Supplement: Figure S6 — Transplantation of SSEA1- dsRed-iPS derived photoreceptor precursor cells induces extensive cellular integration and outernuclear layer repopulation. A–C: Immunocytochemical analysis performed on rho-/- recipient mouse eyes at 21 days post-subretinal injection of SSEA1-negative dsRed-iPS cells targeted against the host donor cell marker dsRed. A–B: Low magnification images used to show the extent of cellular integration post-transplantation. C: High magnification image taken at the outer limit of cellular migration in figure B where sparse cellular integration was observed. This image was taken in an attempt to show detailed donor cell morphology. As shown in these images extensive cellular integration and retinal ONL repopulation was identified across a wide area of the host retina at 3 weeks post-transplantation (A–B). Cells that integrate within the retinal degenerative environment adopt a photoreceptor morphology represented by a single cell body with and outer segment extended toward the RPE and an inner process ending with a synaptic pedicle that extends into the host plexiform layer (C). D–E: Immunocytochemical analysis performed on control rho-/- un-injected contralateral mouse eyes at 21 days post-op against dsRed, ROM1 and recoverin (D) or rhodopsin (E). Contralateral 6–8 week old rhodopsin null mouse eyes do not express ROM1, dsRed or rhodopsin, indicating that rod photoreceptors detected in Rho-/- eye at 21-days post-subretinal injection are iPSC transplant derived. (TIF) [file pone.0018992.s006.tif]

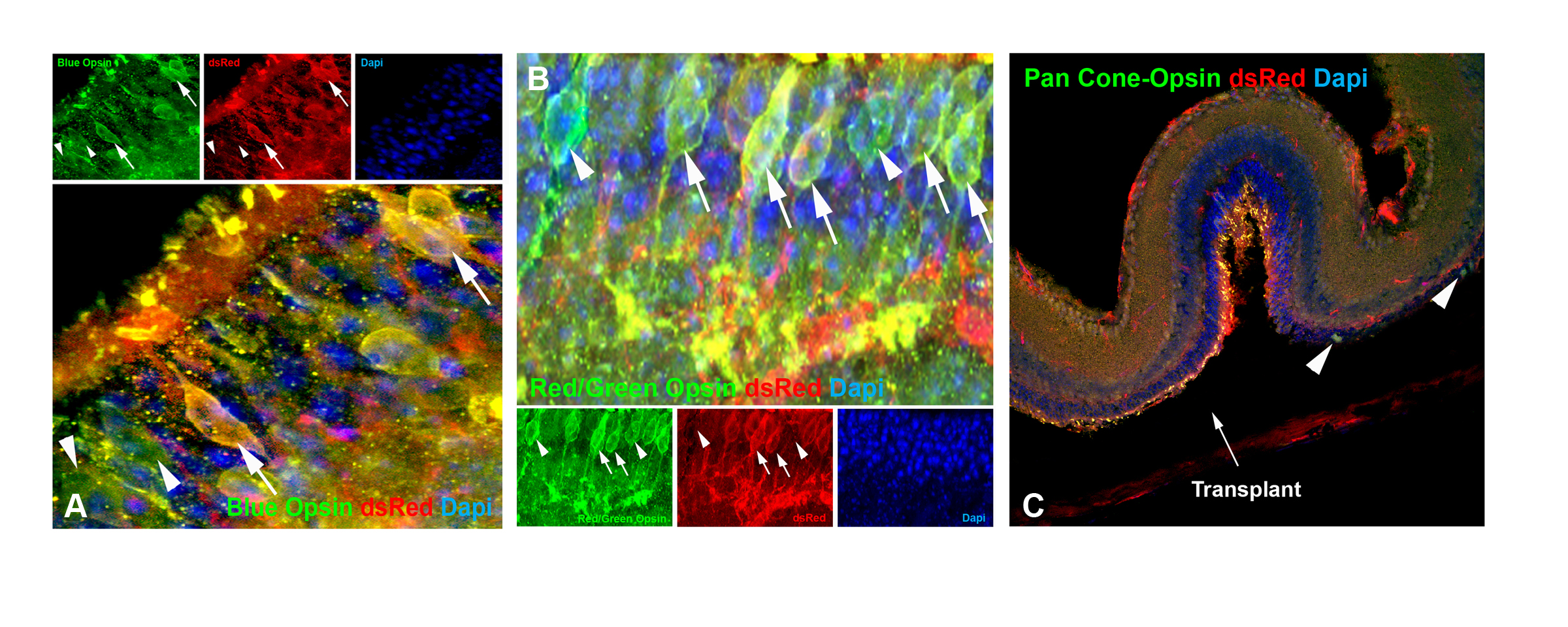

Supplement: Figure S7 — Generation of cone photoreceptors following transplantation of SSEA1- dsRed-iPS derived photoreceptor precursor cells. A–C: Immunocytochemical analysis performed on rho-/- recipient mouse eyes at 21 days post-subretinal injection of SSEA1-negative dsRed-iPS cells targeted against the blue cone photoreceptor marker blue-opsin (A), the red/green cone photoreceptor marker red/green-opsin (B) and the pan cone photoreceptor marker pan-cone-opsin (C). (TIF) [file pone.0018992.s007.tif]

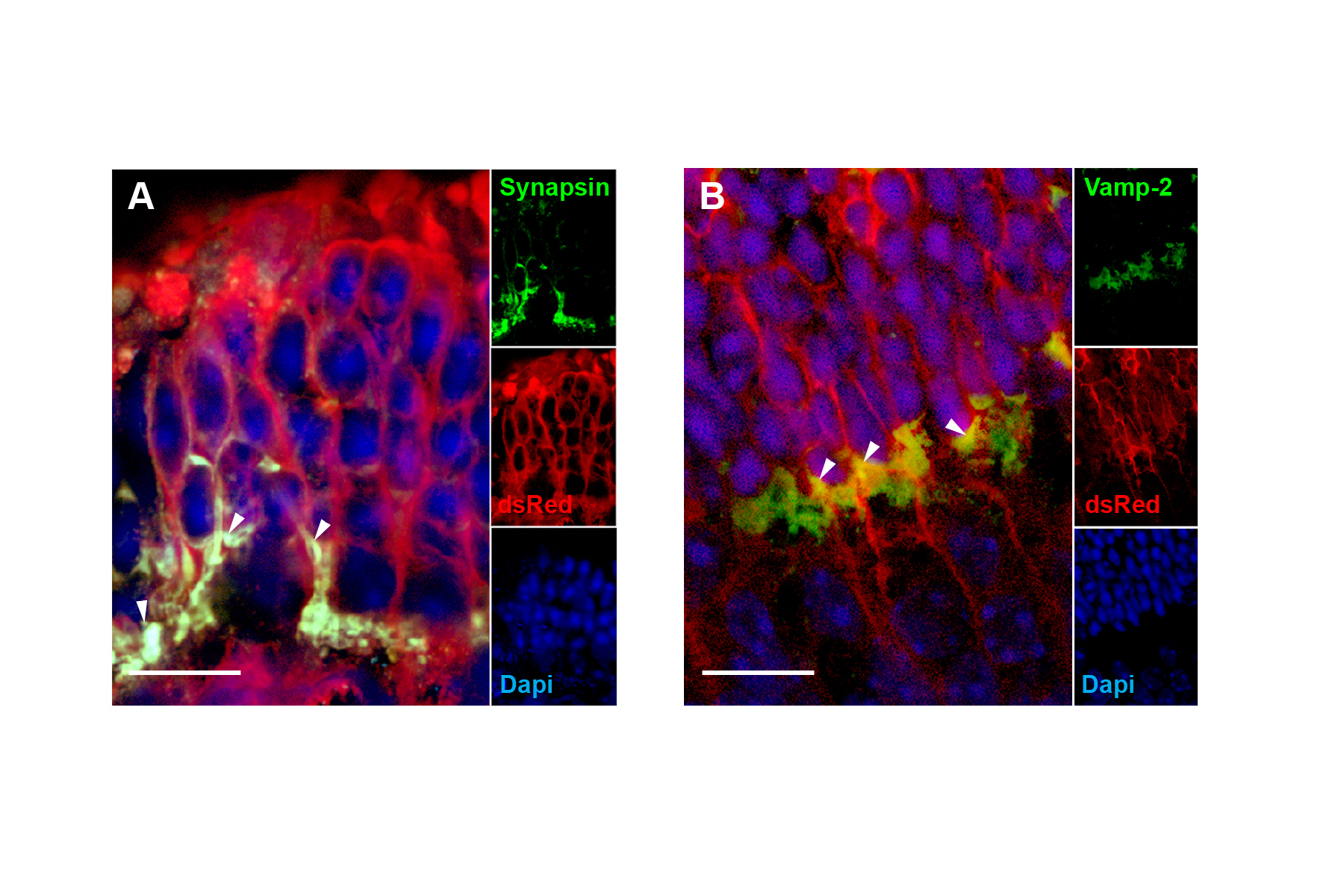

Supplement: Figure S8 — Synaptic integration of dsRed-iPS cell derived photoreceptor precursor cells following subretinal transplantation. A–C: Immunocytochemical analysis performed on rho-/- recipient mouse eyes at 21 days post-subretinal injection of SSEA1-negative dsRed-iPS cells targeted against the synaptic markers synapsin (A) and Vamp-2 (B). Scale bar = 10 µm. Transplanted dsRed-expressing iPS cell derived photoreceptor precursors that integrate into the outer nuclear layer of retinal degenerative mice following subretinal injection form synaptic connections at the level of the outer plexiform layer within the host retina. (TIF) [file pone.0018992.s008.tif]

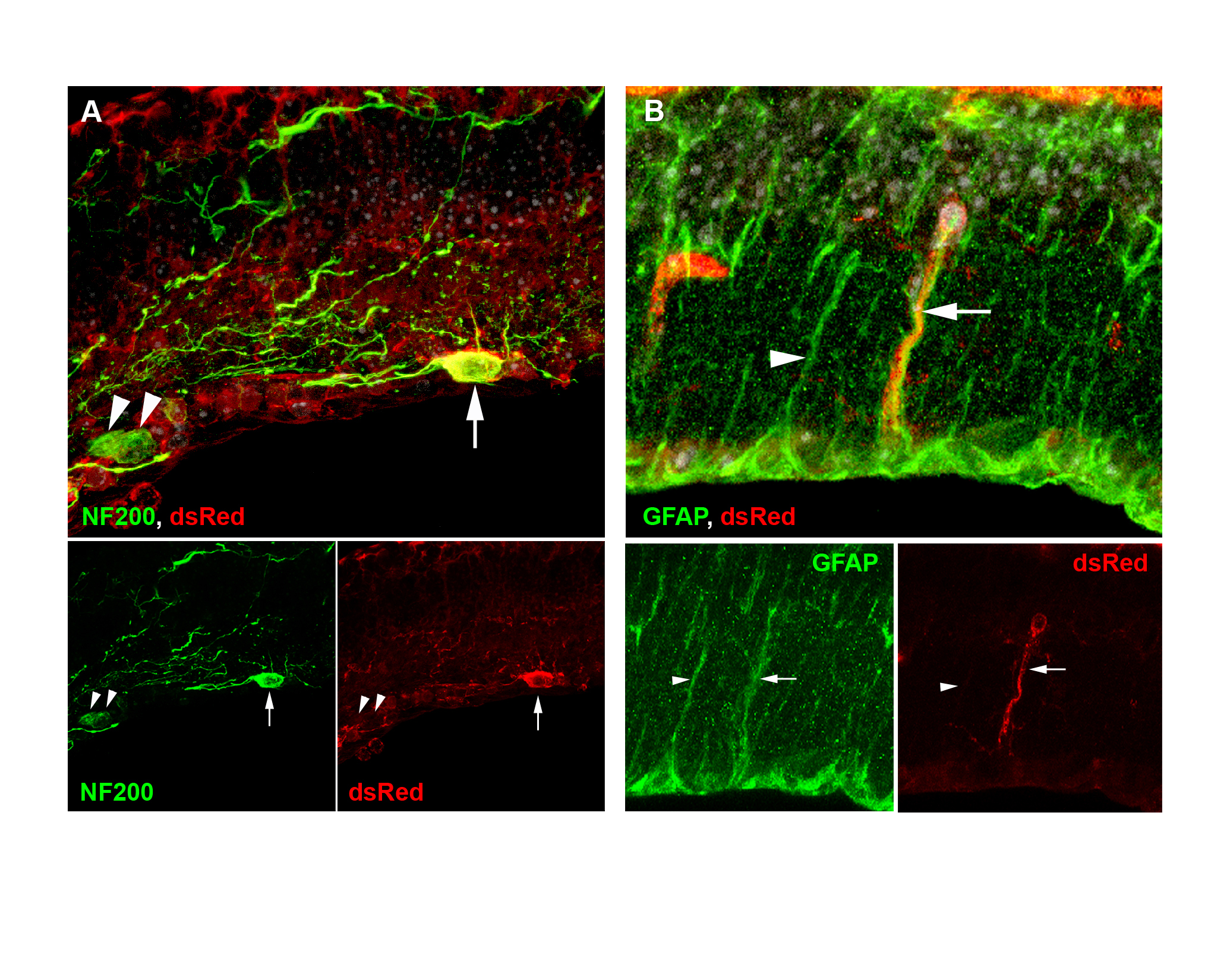

Supplement: Figure S9 — Integration of cell types other then retinal photoreceptors at 21-days post-injection. A-C: Immunocytochemical analysis performed on rho-/- recipient mouse eyes at 21 days post-injection of SSEA1-negative dsRed-iPS cells targeted against the retinal ganglion cell marker NF200 (A) and the glial cell marker GFAP (B). Scale bar = 10 µm. In addition to photoreceptors, transplanted dsRed-expressing iPS cells also gave rise to NF200 expressing retinal ganglion and GFAP-expressing glial cells following ocular injection. Importantly, both cell types took up residence within the appropriate retinal layer and appeared to develop morphologically into the correct cell types. (TIF) [file pone.0018992.s009.tif]
