# Supplementary material for: Transplantation of Adult Mouse iPS Cell-Derived Photoreceptor Precursors Restores Retinal Structure and Function in Degenerative Mice
Source: PLoS One. 2011 Apr 29;6(4):e18992. doi: 10.1371/journal.pone.0018992 (PMC3084746; doi:10.1371/journal.pone.0018992)
Supplement: Table S1 — Gene specific primer sequences used for RT-PCR. (DOC) [file pone.0018992.s010.doc]

| CCCTTCTCCAACGTCACAGG | Rhodopsin Forward |
| --- | --- |
| TGAGGAAGTTGATGGGGAAGC | Rhodposin Reverse |
| GTTCAAGAATCGTAGGGCGAA | CRX Forward |
| GTTCAAGAATCGTAGGGCGAA | CRX Reverse |
| ACGAGCATTCTCAGCAACGTA | Opsin Forward |
| GCCAGGTTGATAATCACTGCAT | Opsin Reverse |
| GCCCTGTCCAAGGAGATCCTG | Recoverin Forward |
| CGCAGAATTTCCTTATTGGCCAG | Recoverin Reverse |
| TTTGGAGGTGGCTGGGTAGATG | NRL Forward |
| ACGATGCTCAGAAGTTTGGGG | NRL Reverse |
| ATCCCAAGGAGCAAGGAGAG | RX Forward |
| TTCTGGAACCACACCTGGAC | RX Reverse |
| AAGGAGGGGGAGAGAACACC | Pax6 Forward |
| TCTGAGCTTCATCCGAGTCTT | Pax6 Reverse |
| TGTTGCCATCAATGACCCCTT | GAPDH Forward |
| CTCCACGACGTACTCAGCG | GAPDH Reverse |
