# Supplementary material for: Transplantation of Adult Mouse iPS Cell-Derived Photoreceptor Precursors Restores Retinal Structure and Function in Degenerative Mice
Source: PLoS One. 2011 Apr 29;6(4):e18992. doi: 10.1371/journal.pone.0018992 (PMC3084746; doi:10.1371/journal.pone.0018992)
Supplement: Methods S1 — Retroviral production and iPS cell generation. (DOC) [file pone.0018992.s011.doc]

**Supplemental Methods**

**Retroviral production and iPS cell generation:** iPS cells were generated from adult dsRed-positive mouse dermal fibroblasts using a protocol previously reported for reprogramming human fibroblasts[[19](#_ENREF_1)] Forced expression of the four transcription factors Oct4, Sox2, Klf4 and c-MYC were induced by retroviral infection of adult dsRed-positive mouse dermal fibroblasts. At 5 days post-infection, cells were passaged onto a monolayer of inactive mouse embryonic fibroblasts and fed daily with pluripotency media (DMEM F-12 media (Gibco) 15% heat inactivated-FBS (Lifeblood Medical Inc.), 0.0008% β-mercaptoethanol (Sigma-Aldrich, St. Louis, MO), 1% 100x NEAA (Gibco), 1x10^6^ units/L of leukemia inhibitory factor (LIF/ESGRO, Millipore, Billerica, MA), 1% penicillin/streptomycin/L-glutamine (Gibco) and 0.2% Fungizone (Gibco)). At 3 weeks post-infection iPS cell colonies were picked, passaged and clonally expanded for further experimentation.

**Focused Microarray Analysis:** To compare iPS cells post-generation to ES cell counterparts for expression of pluripotency markers and to identify retinal progenitor cell/photoreceptor cell production post-differentation a focused microarray analysis was performed. Biotin-tagged cRNA, synthesized from total RNA isolated from undifferentiated D0 and differentiated D33 dsRed-iPS cells was bound to custom made oligo-arrays (containing genes specific to pluripotent stem cells, retinal progenitor cells and retinal neurons) by hybridization at 60°C for 12 to 16 hrs (SABioscience, Frederick, MD) and subsequently imaged using chemiluminescence and x-ray film detection (Kodak/Carestream Health, Bio Max Light Film, Rochester, NY). Films were digitized and uploaded to web-based analysis software (SABioscience) and densitometric data were subsequently analyzed. To control for experimental variation, (e.g., exposure/cRNA loading), densitometric analyses for each probe was corrected against internal housekeeping genes located on each microarray.

**Subretinal Transplantation:** The transplantation procedure used here was performed as described previously[[35](#_ENREF_2)]. Briefly, recipient rho-/- mice were anesthetized by intraperitoneal injection of ketamine (Webster Veterinary Supply) (5mg/kg) and xylazine (Webster Veterinary Supply) (10 mg/kg) and prepared for surgery. 1μl of the previously prepared dsRed-iPS cell suspension (approximately 2-3x10^5^ cells were injected per animal in each of the heterogeneous, SSEA1-depleted and SSEA1-undepleted groups, a depiction of the scale of the transplant has been shown previously by our group[39]) was taken up into a glass micro-needle connected to a 10μl Hamilton syringe (VWR, West Chester, PA) and injected through the sclera into the subretinal space of degenerative recipient eyes (contralateral eyes were used as no surgery controls). Animals were euthanized at 21 days post-op, eyes were enucleated and used in subsequent analysis. No immunosuppression was used for any of the transplantation studies.

**Histology:** Teratomas were fixed in 10% formalin for 24 hours prior to dehydration and caste in rapid polymerizing methyl methacrylate (Technovit 7100, VWR). Samples were removed, mounted on microtome chucks and semi-thin sectioning was performed. H&E staining was performed as per standard protocols.

**SSEA1 Cell depletion:** SSEA1+ dsRed-iPS cells remaining post-differentiation were removed from the culture system prior to subretinal transplantation via magnetic bead-depletion. This procedure was performed in accordance with the manual protocol provided (Miltenyi Biotec, Auburn, CA) and is described in detail in Supplemental Figure 4B.

**Calcium imaging:** 100 D33 SSEA1-negative differentiated iPSCs were mechanically isolated, dissociated, and transferred to a 35 mm glass bottom petri dish (MatTek, Ashland, MA) coated with poly-D-lysine (BD Bioscience), collagen (BD Bioscience), laminin (Gibco) and fibronectin (Sigma-Aldrich), and allowed to adhere at 37°C for 24 hrs prior to recording. Cells were subsequently rinsed with a Ringer's solution maintained at 37°C containing: (mM) NaCl 119, KCl 4.16, CaCl_2_ 2.5, MgCl_2_ 0.3, MgSO_4_ 0.4, Na_2_HPO_4_ 0.5, NaH_2_PO_4_ 0.45 (Sigma-Aldrich), HEPES (Gibco) and glucose (Sigma-Aldrich) at pH 7.4. Cells were then incubated in Ringer's solution containing 0.5 µm fura-2 tetra-acetoxymethyl ester (Fura-2) (Molecular Probes, Carlsbad, CA), 10% pluronic F127 (Sigma-Aldrich) and 250 µm sulfinpyrazone (Sigma-Aldrich) for 40 min at 22°C. Fura-2 was excited by alternating 340 and 380 nm lights and images were obtained every 0.35 sec as a measure of Ca^2+^ concentration. Background intensity was zero. A bolus injection brought the stimulant concentration in the cell bath to 1mM glutamate (Sigma-Aldrich).

**Immunostaining:** Enucleated eyes that had previously received subretinal iPS cell transplants, and cell cultures at various stages of differentiation, were fixed in a 4% paraformaldehyde solution and immunostained as described previously. For teratoma analysis antibodies targeted against ßIII tubulin (Cat# T8660, Sigma-Aldrich), GFAP, (Cat# 18-0063 , Invitrogen, Carlsbad, CA), smooth muscle actin (Cat# ab5694, Abcam, Cambridge, MA) and dsRed (Cat# SC81595, Santa Cruz Biotech, Santa Cruz, CA, Cat# ab62431, Abcam) were employed. To evaluate retinal cell production post iPS cell differentiation primary antibodies targeted against CRX (Cat# SC30150, Santa Cruz Biotech), recoverin (Cat# AB5585, Chemicon, Temecula, CA), rhodopsin (Cat# MAB5316, Chemicon), GS (Cat# AB49873, Abcam), NF200 (Cat# AB1982, Chemicon), and ZO-1 (Cat# 33-910, Invitrogen) were employed. To evaluate the percentage of undifferentiated cells remaining in D33 cultures, antibodies targeted against SSEA1 (Cat# MA1-16907, Thermo Fisher Scientific Inc), Pax6 (Cat# MAB5552, Chemicon, Cat# Pax6, DSHB, Iowa City, IA), and Ki67 (Cat# VP-K451, Vector Laboratories, Burlingame, CA) were employed. To evaluate the level of cellular integration and retinal repopulation antibodies targeted dsRed (Cat# SC81595, Santa Cruz Biotech, Santa Cruz, CA, Cat# ab62431, Abcam), recoverin, (Cat# AB5585, Chemicon), ROM-1 (Cat# PA1-54934, Chemicon), cone opsin (Chemicon, Cat# AB5405, R/G and Cat# AB5407, B), synaptophysin (Cat# M0776, DAKO, Carpinteria, CA), synapsin (Cat# VAP-SV060, Stressgen Bioreagents Corp., Ann Arbor, MI), Bassoon (Cat# MA1-20698, Thermo Fisher Scientific Inc) and VAMP2 (Cat# VAS-SV006, Stressgen Bioreagents Corp.) was employed. Subsequently, Cy2, Cy3 or Cy5-conjugated secondary antibodies were used (Jackson Immunochem, West Grove, PA) and the samples were analyzed using confocal microscopy. Microscopic analysis was performed in such a way that exposure time, gain, and depth of field remained constant between experimental conditions.

**Immunoblotting:** For Western blot analysis, iPS cells taken at various stages of differentiation were homogenized in lysis buffer (50 mM Tris-HCl, pH 7.6, 150 mM NaCl, 10mM CaCl_2_, 1% triton X-100, 0.02% NaN_3,_ (Sigma-Aldrich)), centrifuged, supernatants isolated and protein concentrations determined using a BCA protein assay (Pierce Chemicals, Rockford, IL). Equivalent amounts of protein (50μg) were subjected to SDS-PAGE (8%-10% acrylamide), transferred to nitrocellulose and probed with the following antibodies: CRX (Cat# SC20691, Santa Cruz Biotech), recoverin (Cat# AB5585, Chemicon), rhodopsin (Cat# MAB5316, Chemicon), and ß-actin (Cat# AB20272-100, Abcam; used as a loading control). Blots were visualized with ECL reagents (VWR) and exposed to X-ray film (Kodak). Developed films were subsequently digitized and densitometrically analyzed using the Image J software (each substrate was normalized against β-actin).

**RNA isolation and RT-PCR:** Total RNA was extracted from undifferentiated D0 and differentiated D33 dsRed-iPS cells using the RNeasy Mini-kit (Qiagen, Valencia, CA) following the provided instructions. Briefly, cells were lysed, homogenized and ethanol was added to adjust binding conditions. Samples were spun using RNeasy spin columns, washed and RNA was eluted using RNase-free water. 1 mg of RNA was reverse transcribed into cDNA using the random hexamer (Invitrogen, Carlsbad, CA) priming method and Omniscript reverse transcriptase (Qiagen). All PCR reactions were performed in a 40 mL reaction containing 1x PCR buffer, 1.5 mM MgCl_2_, 0.2 mM dNTPs, 100 ng of DNA, 1.0 U of AmpliTaq Gold (Applied Biosystems, Foster City, CA) and 20 pmol of each gene specific primer. All cycling profiles incorporated an initial denaturation temperature of 94°C for 10 min through 35 amplification cycles (30 sec at 94°C, 30 sec at annealing temperature of each primer and 1 min at 72°C) and a final extension at 72°C for 10 min. PCR products were separated by electrophoresis on 2% agarose gels. Gene specific primers (Invitrogen) are given in Supplemental Table 1.

**Cell Counting:** Cell counts presented in figures 2 and 3 were performed by counting the total number of cells expressing the protein of interest in either the entire heterogenous population (all cells including those immediately adjacent to the originally plated embryoid bodies) or the differentiated population (taken 200um outside of the originally plated embryoid bodies) at 33 days post-differentiation. In each case counts were performed using 10 microscopic fields from each of 3 experimental repeats. As such, statistical analysis was based on counts from 30 microscopic fields.

**Statistical Analysis:** Each experiment was repeated 3 times where appropriate, data are plotted as mean + SEM and significance is noted only if p<0.05, as determined by one-way ANOVA with Tukey testing for post-hoc comparisons or standard students T-test.
